# Genetic control of N-glycosylation of human blood plasma proteins

**DOI:** 10.18699/VJGB-23-29

**Published:** 2023-06

**Authors:** S.Zh. Sharapov, A.N. Timoshchuk, Y.S. Aulchenko

**Affiliations:** MSU Institute for Artificial Intelligence, Lomonosov Moscow State University, Moscow, Russia; MSU Institute for Artificial Intelligence, Lomonosov Moscow State University, Moscow, Russia; MSU Institute for Artificial Intelligence, Lomonosov Moscow State University, Moscow, Russia Institute of Cytology and Genetics of the Siberian Branch of the Russian Academy of Sciences, Novosibirsk, Russia

**Keywords:** glycome, glycans, N-glycosylation, genetics, GWAS, гликом, гликаны, N-гликозилирование, генетика, ПГИА

## Abstract

Glycosylation is an important protein modification, which influences the physical and chemical properties as well as biological function of these proteins. Large-scale population studies have shown that the levels of various plasma protein N-glycans are associated with many multifactorial human diseases. Observed associations between protein glycosylation levels and human diseases have led to the conclusion that N-glycans can be considered a potential source of biomarkers and therapeutic targets. Although biochemical pathways of glycosylation are well studied, the understanding of the mechanisms underlying general and tissue-specific regulation of these biochemical reactions in vivo is limited. This complicates both the interpretation of the observed associations between protein glycosylation levels and human diseases, and the development of glycan-based biomarkers and therapeutics. By the beginning of the 2010s, high-throughput methods of N-glycome profiling had become available, allowing research into the genetic control of N-glycosylation using quantitative genetics methods, including genome-wide association studies (GWAS). Application of these methods has made it possible to find previously unknown regulators of N-glycosylation and expanded the understanding of the role of N-glycans in the control of multifactorial diseases and human complex traits. The present review considers the current knowledge of the genetic control of variability in the levels of N-glycosylation of plasma proteins in human populations. It briefly describes the most popular physical-chemical methods of N-glycome profiling and the databases that contain genes involved in the biosynthesis of N-glycans. It also reviews the results of studies of environmental and genetic factors contributing to the variability of N-glycans as well as the mapping results of the genomic loci of N-glycans by GWAS. The results of functional in vitro and in silico studies are described. The review summarizes the current progress in human glycogenomics and suggests possible directions for further research.

## Abstract (ru)

Гликозилирование является важной модификацией белков, которая влияет как на их физико-химические свойства, так и на выполняемые ими биологические функции. Масштабные популяционные исследования показали, что уровни различных N-гликанов белков плазмы крови ассоциированы с риском развития ряда мультифакторных заболеваний человека. Найденные ассоциации стали основанием для рассмотрения N-гликанов в качестве потенциального источника биомаркеров и терапевтических мишеней. Биохимические пути N-гликозилирования хорошо изучены, однако понимание механизмов общей и тканеспецифической регуляции этих биохимических реакций in vivo весьма ограниченно. Это затрудняет как интерпретацию наблюдаемых ассоциаций уровней N-гликанов с заболеваниями человека, так и разработку биомаркеров и молекулярных мишеней на их основе. Прогресс в области технологий анализа N-гликозилирования белков позволил к началу 2010-х годов проводить исследования регуляции N-гликозилирования с помощью методов генетического анализа, в том числе полногеномного исследования генетических ассоциаций. Применение этих методов дает возможность находить новые, ранее неизвестные регуляторы N-гликозилирования и расширяет представление о роли N-гликанов в контроле мультифакторных заболеваний и комплексных признаков человека. В данном обзоре мы рассматриваем современное состояние исследований генетического контроля популяционной изменчивости уровней N-гликозилирования белков плазмы крови человека. Описаны современные физико-химические методы измерения N-гликомного профиля, приведены базы данных, содержащие гены, вовлеченные в биосинтез N-гликанов. Систематизированы результаты исследований вклада средовых и генетических факторов в популяционную изменчивость N-гликанов, а также результаты картирования геномных локусов N-гликанов методом полногеномного исследования ассоциаций. Представлены результаты последующих функциональных исследо- ваний in vitro и in silico, позволивших предложить новые гены-кандидаты, регулирующие N-гликозилирование белков. В заключение кратко показан текущий прогресс в области гликогеномики человека и описаны возмож- ные пути дальнейших исследований N-гликома.

## Glycomics as a branch of glycobiology

Glycans, also known as poly- or oligosaccharides, are polymers consisting of monosaccharides joined together by glycosidic linkages. Glycans may covalently bind to proteins and lipids by glycosidic bonds to produce glycoproteins and glycolipids, respectively. Glycosylation is one of the most common (Craveur et al., 2014) post- and co-translational protein modifications. It is found that about 20 % of all proteins in nature are glycosylated (Khoury et al., 2011). Meanwhile, over 40 % (by weight) of all proteins in human blood plasma are N-glycosylated (Clerc et al., 2016). Glycosylation affects not only physical and chemical properties of proteins, such as solubility, spatial configuration, folding, etc. (Varki, 1993; Ohtsubo, Marth, 2006; Skropeta, 2009), but their biological function as well. Glycoconjugates, i. e. glycoproteins and glycolipids with covalently bound glycans, are present in cells of all multicellular organisms (Varki, Kornfeld, 2015).

Glycoproteins and glycolipids at the surfaces of cell membranes are involved in various cellular interactions, including cell-cell, cell-extracellular matrix, and cell-macromolecule interactions, as well as interactions between organisms (host-parasite, symbiont-symbiont, etc.) (Ohtsubo, Marth, 2006; Skropeta, 2009; Lauc et al., 2016; Poole et al., 2018), which plays a major part in development and functioning of multicellular organisms (Gagneux et al., 2015).

Numerous studies into the chemical structure of glycans and their metabolism have been carried out in the early 20th century. At the time, however, glycans were primarily considered as structural elements and energy sources for living systems. An explosive development of chemical, physical, and molecular biological methods in glycan research has given birth to a new branch of molecular biology called glycobiology. This domain includes studies of chemical and physical properties of glycans, enzymology of glycan synthesis and degradation, their evolution, mechanisms of glycan recognition by proteins, and the role of glycans in functioning of biological systems, development of human diseases and biological traits, as well as development of new methods for management, prophylaxis, diagnostics, and prediction of diseases (Varki, 2017). Today, glycobiology is a rapidly developing science, and its findings are of great significance for many related fields, including biomedicine and biotechnology (Nikolac Perkovic et al., 2014; Varki, Kornfeld, 2017).

Similarly to genomics, transcriptomics, proteomics, and metabolomics, glycomics is a systematic investigation into glycome, i. e. a variety of all glycans and their contents in a given specimen, whether it be a cell culture, tissue, organ, or the whole organism. The diversity of possible glycoconjugates is beyond imagination. Although the number of monomers incorporated into glycan structure is relatively small, monomers may form various glycosidic bonds to create an abundance of possible glycans. The diversity of glycoconjugates is further increased due to the possibility of a protein having not one, but several glycosylation sites.

In the early XXI century, high-throughput physical and chemical methods were developed, which made it possible to carry out large-scale cohort studies to discover the associations between N-glycome and human diseases and biological traits. Currently, the associations of N-glycans with many multifactorial diseases in humans (Gudelj et al., 2018a; Dotz, Wuhrer, 2019; Reily et al., 2019), including type 2 diabetes and monogenic forms of diabetes (Thanabalasingham et al., 2013; Keser et al., 2017), rheumatoid arthritis (Gudelj et al., 2018b), the Parkinson’s disease (Russell et al., 2017), inflammatory bowel disease (Trbojević Akmačić et al., 2015; Clerc et al., 2018), as well as cardiovascular (Connelly et al., 2016; Wang et al., 2016) and oncological diseases (Fuster, Esko, 2005; Saldova et al., 2014; Mehta et al., 2015; Taniguchi, Kizuka, 2015), have been identified.

The results of observational studies of the associations between protein glycosylation and human diseases do not shed a light on cause-and-effect relationships between them and molecular biological mechanisms underlying these relationships. From 1983 onward, there have been a number of studies into the functional consequences of changes in protein glycosylation (Anthony et al., 2012; Cobb, 2020). Glycosylation of immunoglobulin G (IgG) appears to be the most deeply studied in this regard, due to its importance for adaptive immunity. The IgG molecule has a conserved glycosylation site Asn297, located in the conserved domain CH2 of the heavy chain. This domain plays a major part in binding to Fcγ receptors, which in turn affects the effector function of IgG. It was shown that a decrease in IgG fucosylation level intensified antibody-dependent cellular cytotoxicity (Peipp et al., 2008). The further crystallography investigation demonstrated that the lack of IgG fucosylation led to higher affinity between the Fc domain of IgG and the receptor FcγRIIIA, while the presence of fucose in IgG glycan resulted in steric hindrances during the interactions (Mizushima et al., 2011).

Given the above, a conclusion can be made about the fundamental importance of glycosylation research for the problems of diagnostics, prediction, prophylaxis, and management of human diseases. In 2012, the National Academy of Sciences of the United States presented a report on the necessity of a large-scale glycome study, the reasoning being that glycans are directly involved in pathogenesis of almost all the known diseases (http://www.nap.edu/catalog. php?record_id=13446).

## Structure and diversity of glycans

Carbohydrates are among the main groups of macromolecules identified in biology, along with proteins, lipids, and nucleic acids. The polymerization ability and a large number of chiral atoms allow monosaccharides to form a large variety of stereo- and regioisomers. Four main groups of carbohydrates are determined based on the degree of polymerization as follows: monosaccharides (glucose, fructose, galactose, etc.), disaccharides (molecules consisting of two monosaccharides joined together by a glycosidic bond, e. g. sucrose, lactose, and maltose), polysaccharides with repeating units forming linear or branching compounds (О-antigens of bacteria, amylose, cellulose, and chitin), and glycans, i. e. complex oligosaccharides with non-repetitive units, which can be free or bound to proteins or lipids (glycoproteins, proteoglycans, and glycolipids).

Protein-bound glycans are in turn divided into N-, O-, and C-glycans. N-glycans form a glycosidic bond to the nitrogen atoms (N) of asparagine amino acid; O-glycans – to the hydroxyl groups of serine and threonine amino acids, and C-glycans – to carbon atoms of the tryptophan amino acid. C-glycosylation is rarely observed compared to N- and O- glycosylation (Chauhan et al., 2013).

An important difference between N- and O-glycosylation is that the N-glycosydic bond only forms with the asparagine of the Asn-X-Ser/Thr motif, where “Х” may represent any amino acid, except for proline, whereas no such motif is known for O-glycans. In addition, there is a PNGase F enzyme that specifically cleaves the N-glycosydic bond between a glycan and a protein, which leads to the release of N-glycans into solution for further analysis (Vilaj et al., 2021). As opposed to N-glycosylation, O-glycosylation site does not have a consensus sequence, and the available methods for O-glycan isolation (beta elimination) show lower specificity compared to that for N-glycans (Mulloy et al., 2015). It is part of the reason why technologies and protocols of glycan structure identification and high-throughput analysis are currently more refined for N-glycans, which are the subject matter of this survey.

**Fig. 1.**
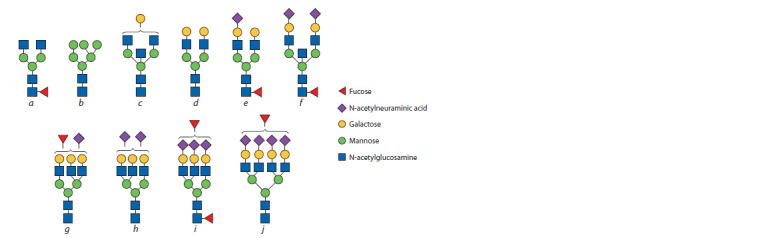
Examples of N-glycan structures: structure b represents a glycan with a large number of mannose residues, while the other glycans have a complex structure. Structures a, c, d–f are biantennary glycans, g–i are triantennary glycans, and structure j is a tetraantennary glycan. N-glycan backbone fucosylation is seen in structures a, e, f, and i; antennary fucosylation (fucose is bound to an antenna) is seen in structures g, i, and j. A bisecting N-acetylglucosamine (GlcNAc) residue is seen in structures c and f. Galactose and N-acetylneuraminic acid residues are shown in structures c–j and e–j respectively.

The most common monomers in N-glycans include monosaccharides, such as mannose, fucose, galactose, N- acetylglucosamine (GlcNAc), and N-acetylneuraminic acid (Fig. 1). N-glycan structure always includes a backbone (Manα1-3(Manα1-6)Manβ1-4GlcNAcβ1-4GlcNAcβ1- Asn-X-Ser/Thr) the other monomers bind to by glycosidic linkages. Glycans may have a branching structure with one or more branches called antennae. Monomers, such as galactose, N-acetylneuraminic acid, or fucose, can bind to any of the antennae. Fucose can also bind directly to the backbone. Negatively charged N-acetylneuraminic acid is the only monomer in N-glycans that carries a charge. The glycans not containing N-acetylneuraminic acid are neutrally charged. N-acetylneuraminic acid in N-glycans is always bound to a galactose residue

The Oxford glycan notation, one of the most commonly used glycan nomenclatures (Harvey et al., 2009), operates as follows:

1. The letter “F” at the very beginning of the name indicates the presence of fucose bound to the backbone

2. It is followed by the “AN” sequence, where N is the number of antennae (branches) in the glycan structure.

3. Then, if the sugar backbone is bisected, the letter “B” (bisecting) is added.

4. If antennary branches are fucosylated, the letter “F” is added.

5. If the glycan structure includes galactose bound to one or several antennae, then the sequence G[n1,n2,...]N follows, where N is the number of galactose residues in a glycan, and n1 indicates the carbon atom of galactose, with which glycosidic bond is formed.

6. If the glycan structure includes N-acetylneuraminic acid bound to one or several antennae, then the sequence S[n1,n2,...]N follows, where N is the number of N-acetylneuraminic acid residues in a glycan, and n1 indicates the carbon atom of N-acetylneuraminic acid, with which glycosidic bond is formed.

For example, the designation FA2 shows the presence of a fucose residue bound to the backbone and two antennae in the glycan structure. The designation A3BG3S1 shows that glycan structure includes three antennae, sugar backbone bisection, three-antenna galactosylation, and one-antenna sialylation

## Genes involved in biological pathways of N-glycosylation

As opposed to mRNA and proteins encoded in the genomic DNA sequence and synthesized as a result of matrix processes, a glycan structure is not encoded in the genome directly, and its biosynthesis is a branching network of biochemical reactions (Lombard et al., 2014). The final structure of a glycan is determined by the interaction of a multitude of molecules and factors, including substrates and their accessibility, the enzyme activity associated with glycan biosynthesis and degradation, their localization and competition for substrate, and transport proteins (Kukuruzinska, Lennon, 1998; Nairn et al., 2008, 2012; Moremen et al., 2012). It was also shown that structure and diversity of N-glycans present in specific cells and tissues is partially regulated at the level of gene transcription encoding the proteins involved in glycan synthesis and degradation (Nairn et al., 2008, 2012; Moremen et al., 2012).

Glycan biosynthesis occurs in the endoplasmic reticulum (ER) and Golgi apparatus (GA). The KEGG database currently includes the data on over 300 enzymes involved in glycan synthesis and degradation processes (Kanehisa et al., 2017). Glycosyltransferases transporting activated monosaccharides to growing glycans are among the key enzymes directly involved in N-glycan biosynthesis. The dedicated CAZy database (Lombard et al., 2014) provides annotation and classification for over 200 glycosyltransferases, at least 40 of them associated with the protein N-glycosylation pathway. In addition, K.S. Egorova et al. developed the CSDB_GT database (Egorova et al., 2021) including the experimentally confirmed CAZy activities. N-glycan biosynthesis stages and the respective glycosyltransferase genes are described in detail in surveys (Saito, Ishii, 2002; Mohanty et al., 2020).

## Physical and chemical methods for high-throughput N-glycan sequencing

**Fig. 2.**
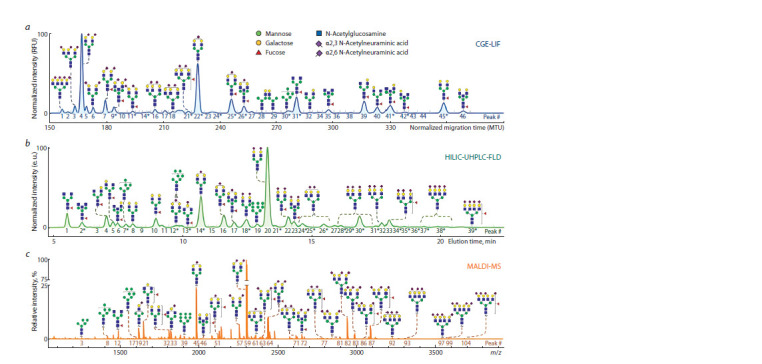
N-glycosylation profiles of blood plasma proteins obtained using UHPLC, xCGE-LIF, and MALDI-MS. N-glycome profile obtained using: a – xCGE-LIF (Reiding et al., 2019); b – UHPLC (Reiding et al., 2019; Zaytseva et al., 2020); c – MALDI-MS after differential esterification of N-acetylneuraminic acid (Vreeker et al., 2018; Reiding et al., 2019; Zaytseva et al., 2020). Modified after (de Haan et al., 2022).

Rapid development of glycobiology combined with the huge success of epidemiological population studies boosted the development of high-throughput glycome profiling methods for blood plasma proteins. In the recent decade, several highthroughput N-glycome profiling methods have been developed (Huffman et al., 2014), specifically high and ultra-high performance liquid chromatography (HPLC and UHPLC), multiplex capillary gel electrophoresis with laser-induced fluorescence detection, (xCGE-LIF), liquid chromatography electrospray mass spectrometry (LC-MS), matrix-assisted laser desorption/ionization time-of-flight mass spectrometry (MALDI-TOF-MS). Representative N-glycosylation profiles of human blood plasma proteins obtained using three different methods are presented in Fig. 2.

A detailed comparison of the five most common N-glycome profiling methods for human blood plasma proteins is presented in the review (de Haan et al., 2022). Despite the differences in technologies, all these methods include several key stages, such as sample preparation (cell culture, tissue, organ, or organism), N-glycan isolation (for example by cleaving from glycoconjugates), separation of N-glycans and content measurement (absolute and relative values) (Huffman et al., 2014).

Each of these glycan analysis methods has its advantages and shortcomings. UHPLC and xCGE-LIF are used to analyze glycans cleaved from proteins, while MALDI-TOF-MS and LC-MS based on mass spectrometry make it possible to analyze glycopeptides with the protein regions containing covalently bound glycans, which provides valuable information on glycosylation of specific proteins. Compared to UHPLC and xCGE-LIF, MALDI-TOF-MS and LC-MS perform better with regard to distinguishing glycans with different molecular weights, but are unable to distinguish glycan stereoisomers. UHPLC and xCGE-LIF provide more accurate quantitative estimates due to their high resolution. In addition, they are characterized by high performance and lower initial costs compared to the methods based on mass spectrometry.

UHPLC has turned out to be the most popular highthroughput N-glycome profiling method for blood plasma proteins among the listed above (Akmačić et al., 2015) due to its relative cheapness, improved resolution (compared to HPLC), and high performance. By the time this review was composed (October 2022), human blood plasma glycome had been studied in about 200,000 samples all over the world, with about 80 % of the samples studied using UHPLC (G. Lauc, personal message).

## Heritability of N-glycosylation levels of human blood plasma proteins

By the early 2010s, the development of high-throughput glycome profiling and genetic analysis methods had made it possible to carry out the first research efforts in genetic control of glycosylation based on the findings of cohort studies. There were a number of reasons why N-glycome of blood plasma became the main research focus: first, compared to other human tissues, blood plasma is a more accessible subject matter; second, as said above, the technologies for N-glycan level measurement and structure identification were more refined. The most common glycoproteins studied in human blood plasma were immunoglobulins G, A, and M, fibrinogen, transferrin, haptoglobin, etc. (Clerc et al., 2016), while liver cells and antibody-producing cells were the main glycoprotein source (Uhlén et al., 2015; Clerc et al., 2016).

Population variability of human blood plasma glycans, their heritability (trait dispersion due to genetic differences), as well as the effects of various environmental factors on glycan levels were studied in (Knezević et al., 2009). Glycan levels were measured using HPLC. The authors of the paper made several major conclusions. First, high population variability of glycosylation levels was discovered. Second, the authors discovered the significant effect biological sex and age had on glycan levels. Third, heritability of glycan levels varied (the average heritability index h2 = 34.7 % and the standard deviation of 15.5 %), which implies that glycans were controlled by both genetic and environmental factors.

In (Zaytseva et al., 2020), the authors assessed the heritability of 39 N-glycan traits measured using UHPLC. It was shown that the heritability was over 50 % (average heritability index h2 = 48.0 % and the standard deviation of 17.7 %) for 24 out of 39 traits, which confirmed the hypothesis on the significant effect both environmental and genetic factors had on blood plasma glycome. In (Clerc et al., 2016; Uhlén et al., 2019), the authors pointed out the highest heritability (> 50 %) in biantennary glycans with backbone fucosylation and reduced sialylation of antennary chains included in immunoglobulins, primarily IgG being the most common glycoprotein among all human blood plasma proteins. Average and high heritability (30–62 %) was observed in bi- and triantennary glycans with high sialylation of antennary chains. In (Jain et al., 2011), the authors assumed that high heritability in this case might be explained by the presence of these structures in a large number of glycoproteins (transferrin, hemopexin, alpha- 1-antitrypsin, alpha-1-acid glycoprotein) causing errors in estimating genetic factors for each of them in isolation, and by the fact that these glycans were primarily contained in glycoproteins synthesized by liver cells, specifically acute-phase proteins, the glycosylation of which was significantly affected by the environment

Despite the fact that heritability studies have made it possible to estimate the portion of trait variability controlled by genome, they have not revealed specific genomic regions affecting the manifestation of traits. The latter may be found using quantitative trait gene mapping methods, in particular genome-wide association studies

## Genome-wide association studies of N-glycan levels associated with blood plasma proteins

Genome-wide association study (GWAS) is the most common method for mapping loci of human diseases and complex traits. This method implies the analysis of associations between a large number (hundreds of thousands to tens of millions) of genetic markers distributed across the whole genome and the studied trait. Typically, large samples (several thousand to millions) of species or individuals are analyzed. The availability of these data makes it possible to essentially test the whole genome for associations with the studied trait and find new previously undiscovered associations between loci and traits. GWAS studies are usually designed around several samples. The findings from samples are combined using genome-wide meta-analysis techniques (Winkler et al., 2014), which increases the total sample size and the statistical power of the association analysis

**Fig. 3.**
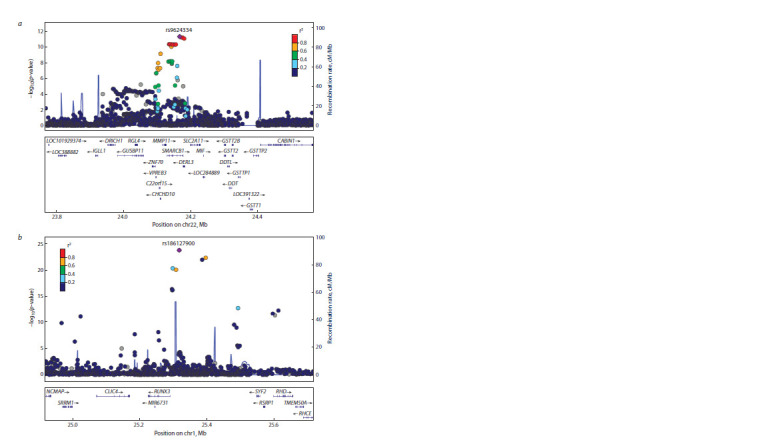
Examples of regional association plots visualizing the association between the trait and genetic markers in the locus. A negative decimal logarithm of the p-value is plotted on the Y-axis. Genomic coordinates of the genetic marker (SNP) are plotted on the X-axis. The association signal may be located in the encoding region of several genes (a) or may not include any genes at all (b).

The presence of the association between a genomic locus and the studied trait does not by itself clarify the molecular biological mechanism underlying the discovered association. The discovered loci may contain from one to tens of genes, but they can also include none (Fig. 3) (Visscher et al., 2012, 2017). There is a multitude of reasons why an association can occur, i. e. the presence of encoding substitutions in the locus affecting the structure and functioning of the gene product (protein or RNA) or the presence of substitutions affecting the specificity of binding between transcription factors and regulatory regions. The number of functional variants may vary from one to many (Yang et al., 2012).

Identification of functional genes in the discovered loci and the mechanisms of their effect on the studied traits is the critical problem of functional studies performed using molecular and cellular biology methods. Here, the number of possible hypotheses to be tested grows geometrically (in theory) depending on the number of possible molecular association mechanisms. Taking into account the complexity, expensiveness, and labor intensity of molecular and cellular biological methods, primary bioinformatic prioritization of hypotheses on the association mechanisms becomes extremely important. Numerous methods for in silico functional annotation have been developed (Yang et al., 2012; Bulik-Sullivan et al., 2015; Pers et al., 2015; McLaren et al., 2016; Staley et al., 2016; Zhu et al., 2016; Pasaniuc, Price, 2017; Hemani et al., 2018) making it possible to prioritize the hypotheses on association mechanisms, thereby increasing the efficiency of future molecular and biological research.

The subject matter in the available studies of genetic control of glycosylation using the GWAS approach was as follows: the total N-glycome of human blood plasma proteins (the subject matter of this review) (Lauc et al., 2010a, b; Huffman et al., 2011; Sharapov et al., 2019, 2020), N-glycome of immunoglobulin G, i. e. the most common N-glycoprotein in blood plasma (Lauc et al., 2013; Shen et al., 2017; Wahl et al., 2018; Klarić et al., 2020; Shadrina et al., 2021), and N-glycome of transferrin (Landini et al., 2022) secreted by liver.

At present, the results of five GWAS studies of the total N-glycome of human blood plasma proteins are available (Lauc et al., 2010a, b; Huffman et al., 2011; Sharapov et al., 2019, 2020).

## GWAS studies of the total N-glycome of human blood plasma proteins

The first GWAS studies into N-glycosylation levels of human proteins were performed in 2010–2011 (Lauc et al., 2010a, b; Huffman et al., 2011). The authors used HPLC to analyze glycosylation levels, and the marker density of the genetic data was relatively low by today’s standards at up to 2.5 million SNPs per genome. Six loci (FUT8, HNF1A, FUT3/FUT5/FUT6, MGAT5, B3GAT1, SLC9A9) associated with N-glycosylation of human blood plasma proteins were identified in these GWAS studies. It should be noted that none of the studies used independent samples to confirm the results.

The study published in 2019 (Sharapov et al., 2019) used the data from the TwinsUK Registry (Spector, Williams, 2006; Moayyeri et al., 2013). The genome-wide genotyping data and the primary UHPLC data on the N-glycome of blood plasma proteins were available for 2763 participants. The SNP number was increased from 2.5 to 8.5 million by means of imputation using the data of the 1000 Genomes Project and the appropriate quality control. As a result, the association was confirmed for 5 out of 6 previously identified loci (except for SLC9A9), and 10 new loci were discovered.

Based on four studies (Lauc et al., 2010a, b; Huffman et al., 2011; Sharapov et al., 2019), associations with 16 loci were found, with 15 of them confirmed later in (Sharapov et al., 2020) (see the Table) using the largest (at the time of the study) collection of genomic and glycomic data for 4802 specimens from four samples, namely EPIC-Potsdam, PainOmics, SOCCS, and SABRE, described in detail in the appendices (Sharapov et al., 2020). To put it briefly, the participants of the aforementioned studies were genotyped using the following DNA chips: EPIC-Potsdam (Human660W, 560,000 SNP, HumanCoreExome, 410,000 SNP, InfiniumOmniExpressExome, 850,000 SNP), PainOmics (Illumina HumanCore BeadChip, 720,000 SNP, Illumina GSA, 300,000 SNP), SOCCS (HumanHap300/Human- Hap240S, 510,000 SNP), SABRE (Illumina Human Core Bead Chip, 330,000 SNP).

**Table 1.**
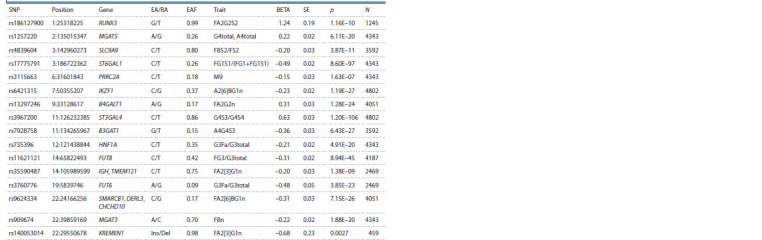
Loci the associations of which were confirmed using independent samples (except for KREMEN1) in (Sharapov et al., 2020) Notе. EA/RA – effect allele/reference allele; EAF – effect allele frequency; BETA/SE – effector allele effect on a trait and its standard error.

EPIC-Potsdam cohort study included 27,000 participants at the ages from 35 to 65, who were selected randomly from the population of the city of Potsdam (Germany) in the years from 1994 to 1998 (Boeing et al., 1999). PainOmics (Allegri et al., 2016) was the case-control study aimed at finding potential biomarkers for dorsalgia and therapeutic targets for its management. The sample of 3400 participants including the residents of Italy, Belgium, England, and Croatia was composed in the years from 2014 to 2016.

The Scottish project SOCCS (Theodoratou et al., 2016; Vučković et al., 2016) was the case-control study aimed at investigating the risk factors of colorectal cancer. The data on 2000 colorectal cancer patients and 2100 control subjects were collected in the research. SABRE is the population study initiated in 1988 (Tillin et al., 2012). Overall, the data on 4800 participants aged from 40 to 69 residing in West London (Great Britain) were collected.

To prioritize new protein glycosylation regulator genes in the confirmed loci and pose hypotheses on potential mechanisms at work in these loci, the authors of (Sharapov et al., 2019) used a combination of quantitative genetics and bioinformatics methods and approaches as follows

1. Gene prioritization based on the results of eQTL colocalization analysis. Colocalization methods, particularly the SMR/HEIDI method (Zhu et al., 2016) used by the authors, made it possible to identify genes, the changes in the expression of which (at the mRNA level) mediated the association between SNPs and the studied trait.

2. Gene prioritization based on the determination of possible functional consequences of nucleotide substitutions with high SNP linkage disequilibrium associated with N-glycome traits. The VEP (McLaren et al., 2016), FATHMM-XF (Rogers et al., 2018), and FATHMM-InDel (Ferlaino et al., 2017) methods were used to select SNPs, where substitutions changed the primary amino acid sequence of a protein and/or were recognized as pathogenic. Genes with sequences affected by said substitutions were prioritized as candidate genes.

3. Gene prioritization based on their involvement in various biological pathways. The DEPICT method (Pers et al., 2015) prioritized genes and biological pathways based on the results of enrichment analysis (overrepresentation of genes pertaining to specific biological pathways in the associated loci), which in turn was performed based on the pre-calculated probability of involvement of a specific locus in a particular gene network and/or biological pathway.

If the methods above failed to achieve gene prioritization for a certain locus, then the gene closest to the SNP with the most significant association in the locus was selected.

## Candidate genes involved in N-glycosylation of blood plasma proteins

**Fig. 4.**
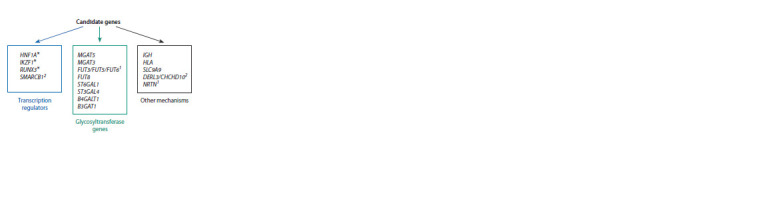
Candidate genes regulating N-glycosylation levels of human blood plasma proteins suggested in (Sharapov et al., 2019). The asterisks indicate the genes experimentally confirmed to be involved in N-glycosylation regulation. The superscripts indicate prioritization of candidate genes within the locus.

As a result of in silico studies within the investigation of the total N-glycome of blood plasma (Sharapov et al., 2019), 20 candidate genes were prioritized for 15 loci (Fig. 4).

The detailed description of these genes and the hypotheses on their possible roles in N-glycosylation regulation of human blood plasma proteins are presented in this section.

The genes coding for glycosyltransferase enzymes involved in N-glycan biosynthesis emerge as candidate genes in 8 loci (MGAT5, MGAT3, FUT3/FUT5/FUT6, FUT8, ST6GAL1, ST3GAL4, B4GALT1, B3GAT1) out of 15.

MGAT5 coding for GnT-V enzyme, i. e. alpha-1,6-mannosylglycoprotein 6-beta-N-acetylglucosaminyltransferase, is the candidate gene in the locus on the second chromosome, 125 Mbp. This enzyme transports the N-acetylglucosamine residue to the mannose of N-glycan, which produces a tri- or tetraantennary N-glycan. The locus with MGAT5 showed an association with glycomic traits reflecting tri- and tetraantennary glycan levels (Sharapov et al., 2019).

MGAT3 coding for N-acetylglucosaminyltransferase GnT-III, i. e. beta-1,4-mannosylglycoprotein 4-beta-N- acetylglucosaminyltransferase, is the candidate gene in the locus on the 22nd chromosome, 39 Mbp. This enzyme transports the N-acetyl glucosamine residue to the mannose of N-gly-can so as to produce backbone bisection. The pleiotropic effect of this locus on both N-glycan levels and MGAT3 expression in CD19+ cells (B-lymphocytes) was demonstrated (Sharapov et al., 2019; Klarić et al., 2020).

FUT8 coding for Fuc-TVIII enzyme, i. e. alpha-(1, 6)-fucosyltransferase, is the candidate gene in the locus on the 14th chromosome, 66 Mbp. This enzyme transports fucose residue to N-acetylglucosamine of the N-glycan backbone, and through that is responsible for N-glycan backbone fucosylation. It is worth noting that loci FUT8 and MGAT3 showed association with traits FBS2/(FS2+FBS2) and FBS2/ FS2 reflecting the presence of backbone bisection in biantennary glycans with backbone fucosylation (Sharapov et al., 2019), which is consistent with the known phenomenon of interference of Fuc-TVIII and GnT-III enzyme activities (Brockhausen, Schachter, 1996).

FUT6, FUT5, FUT3, and NRTN are candidate genes in the locus on the 19th chromosome, 5.8 Mbp. NRTN codes for a neurotrophic factor regulating neuron survival and functioning. FUT6 and FUT3/FUT5 code for Fuc-TVI and Fuc-TIII enzymes, i. e. fucosyltransferase 6 and 3, respectively, transporting fucose residue from the GDP-fucose to the N-acetylglucosamine by forming an alpha-1,3(4)-glycosydic bond. These enzymes are responsible for antennary fucosylation of N-glycans. In (Sharapov et al., 2019), it was shown that this locus is associated with antennary fucosylation in tri- and tetraantennary glycans. It is of note that rs17855739 SNP is located in FUT6. This SNP codes for G>A substitution (А allele frequency in human populations is about 12 %, according to the TopMED database), which leads to the replacement of negatively charged glutamic acid with positively charged lysine at position 247 (p.Glu247Lys). This substitution is located in the catalytic domain of Fuc- TVI enzyme and causes enzyme inactivation, and therefore this variant may have functional effect on glycosylation of human blood plasma proteins. It should be mentioned that FUT3, FUT5, and FUT6 descend from a common ancestral gene as a result of two duplications (Dupuy et al., 2002). In addition, FUT5 expression at the transcription and translation level in a human organism is much weaker compared to FUT3 and FUT6 (Taniguchi et al., 2014).

ST6GAL1 is the candidate gene in the locus on the third chromosome, 186 Mbp. ST6GAL1 codes for alpha-2,6-sialyltransferase 1. This enzyme catalyzes the formation of the alpha-2,6-glycosydic bond between N-acetylneuraminic acid and N-acetylglucosamine bound to galactose of N-glycan. The ST6GAL1 locus showed the association with the levels of mono- and disialylated N-glycans and their precursors (Sharapov et al., 2019).

ST3GAL4 is the candidate gene in the locus on the 11th chromosome, 126 Mbp. ST3GAL4 codes for alpha-2,3-sialyltransferase enzyme transporting the N-acetylneuraminic acid residue. This locus showed an association with the levels of various sialylated N-glycans (Sharapov et al., 2019).

B4GALT1 is the candidate gene in the locus on the 9th chromosome, 33 Mbp. B4GALT1 codes for galactosyltransferase enzyme binding galactose to various substrates, including N-acetyl glucosamine. The B4GALT1 locus was associated with the levels of galactosylated biantennary N-glycans and their precursors (Sharapov et al., 2019). It is also known that a series of mutations in B4GATL1 leads to a congenital disorder of glycosylation (Staretz-Chacham et al., 2020).

B3GAT1 coding for galactosylgalactosylxylosylprotein- 3-beta-glucuronosyltransferase 1 enzyme is the candidate gene in the locus on the 11th chromosome, 134 Mbp. This enzyme catalyzes the transport of glucuronic acid in HNK-1 epitope biosynthesis. This epitope is expressed on lymphocytes, but its presence on blood plasma proteins remained undiscovered for some time. The association of this locus with N-glycan levels in blood plasma proteins was first shown in (Huffman et al., 2011). The presence of glucuronic acid in N-glycome of blood plasma, which can explain the association of the locus, was discovered in (Sharapov et al., 2019).

Candidate genes in seven other loci are not glycosyltransferase genes. Three genes, SMARCB1, DERL3, and CHCHD10, were prioritized in the locus on the 22nd chromosome, 39 Mbp. The strongest association signal in the locus is observed in the coding sequence of SMARCB1 gene. SMARCB1 codes for the protein of hSWI/SNF complex acting as a chromatin remodeler. SMARCB1 gene product plays a major part in carcinogenesis inhibition, cell proliferation and differentiation (Pottier et al., 2007).

DERL3 codes for the enzyme involved in the degradation of luminal glycoproteins with incorrect tertiary structure in the endoplasmic reticulum (Oda et al., 2006). The pathogenic variant rs3177243 is also found in this locus, in the coding sequence of DERL3 gene

CHCHD10 codes for mitochondrial protein observed in fibrils of mitochondrial cristae. It was shown that genetic association of this locus with N-glycan levels in proteins may be mediated by the effect of nucleotide substitutions on CHCHD10 expression in blood cells (Sharapov et al., 2019). The direct involvement of mitochondrial proteins in glycosylation processes remained undiscovered before 2017, when the paper showing the role of mitochondrial fragmentation and the number of ER-mitochondria contacts in representation of sialylated glycans on the surface of glioblastoma cells, which in turn affected glioblastoma cell recognition by lymphocytes, was published (Martinvalet, 2018).

The locus on the 14th chromosome, 105 Mbp, contains the IGH gene cluster coding for heavy chains of immunoglobulins. IgG is the most common N-glycoprotein in human blood plasma (Clerc et al., 2016), and its constitutive N-glycosylation site is located in the heavy chain

SLC9A9 is the candidate gene in the locus on the third chromosome, 142 Mbp. SLC9A9 codes for the Na+/H pump, presumably regulating the pH level in the Golgi apparatus (GA). Protein glycosylation occurs in the GA, and, according to the available data, it is a pH-sensitive process (Kellokumpu, 2019). The processes in the GA affect the synthesis of heterodimeric complexes responsible for glycosylation (Hassinen et al., 2011). It was shown in (Rivinoja et al., 2009) that a pH increase in the GA may disrupt terminal N-glycosylation (including sialylation) due to incorrect localization of glycosyltransferases. In accordance with this hypothesis, the SLC9A9 locus showed an association with tetra-sialylated N-glycan levels in (Huffman et al., 2011) and with sialylated N-glycan levels in (Sharapov et al., 2019).

HNF1A is the candidate gene in the locus on the 12th chromosome, 121 Mbp. A detailed functional study into this locus in (Lauc et al., 2010a) showed that HNF1A coding for the hepatocyte transcription factor regulates the expression of most fucosyltransferase encoding genes, FUT3, FUT5, FUT6, FUT8, FUT10, and FUT11, in the HepG2 cell line obtained from liver cells. The same study demonstrated that HNF1A regulates the expression of genes encoding the key GDP-fucose synthesys enzymes, and GDP-fucose acts as a substrate for fucosyltransferases. This implies that HNF1A plays a major part in glycan fucosylation processes.

IKZF1 is the candidate gene for the locus on the 7th chromosome, 50 Mbp. It was shown earlier (Lauc et al., 2013) that this locus was associated with IgG glycosylation, and IKZF1 was suggested as the candidate gene for the locus. IKZF1 encodes the DNA-binding protein Ikaros, a transcription regulator involved in chromatin remodeling. It is of note that the IKZF1 locus showed the association with levels of N-glycans with backbone fucosylation in blood plasma proteins, with which the FUT8 locus was associated (Sharapov et al., 2019). IKZF1 is considered as an important lymphocyte differentiation regulator (Sellars et al., 2009; Marke et al., 2018).

Since IgG-secreting cells are lymphocyte derivatives, IKZF1 gene was selected as the candidate gene in the locus, and the hypothesis on its role in regulation of backbone fucosylation in IgG N-glycans through FUT8 expression regulation was posed (Sharapov et al., 2019). In addition, it was experimentally shown in (Klarić et al., 2020) that IKZF1 knockdown in MATAT6 IgG-secreting cells leads to more than tripled FUT8 expression and increased fucosylation level in the secreted IgG.

RUNX3 is the candidate gene in the locus on the first chromosome, 25 Mbp. This gene codes for Runt domaincontaining protein, a transcription factor, which, similarly to IKZF1 (Sellars et al., 2009), plays a major part in B-lymphocyte maturation and differentiation.

The candidate genes for the HLA locus (human major histocompatibility complex) on the sixth chromosome, 25–32 Mbp, are not presented due to a high chance of false positive. The HLA locus is unique in terms of quantitative genetics of multifactorial human traits (Kennedy et al., 2017). This locus shows the highest gene density in the human genome; it also demonstrates the highest degree of polymorphism at the nucleotide level; locus alleles show high linkage disequilibrium throughout the whole locus spanning 8 Mbp.

## Gene regulatory network of N-glycosylation of human blood plasma proteins

**Fig. 5.**
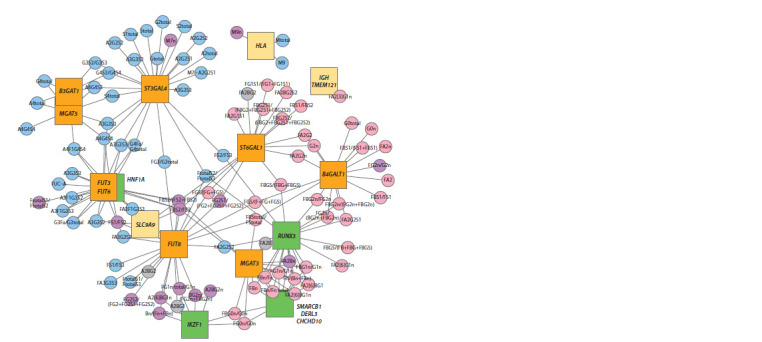
A network view of associations between loci and glycan traits The squares indicate the loci; each locus is presented with the names of prioritized genes. The loci with prioritized glycosyltransferase genes are highlighted in orange, and the loci with prioritized transcription factor genes – in green. The circles represent glucan traits. Glycan names in circles are given in accordance with the Oxford notation (see the section “Structure and diversity of glycans”). Blue indicates N-glycans linked to glycoproteins secreted by hepatocytes. Pink indicates N-glycans linked to glycoproteins (specifically immunoglobulins) secreted by lymphocyte cells. Purple indicates N-glycanslinked to both glycoproteins secreted by hepatocytes and glycoproteins secreted by lymphocyte cells. Gray indicates N-glycans for which classification was not performed. The links in the network indicate genetic associations with p-value < 2.67 ∙ 10–5.

The recent studies into N-glycome of blood plasma (Sharapov et al., 2019, 2020) have demonstrated a significant association of 15 loci with 116 out of 117 glycan traits. In total, significant association has been shown by 214 locustrait pairs. These data were used in (Sharapov et al., 2019) to reconstruct the gene regulatory network of N-glycan levels in blood plasma proteins (Fig. 5). This network visualizes the association between the discovered loci and N-glycan levels in blood plasma proteins.

To build the network, glycomic traits were classified into four groups based on the tissue secreting N-glycoproteins into blood plasma. The first group included the traits reflecting N-glycan levels in immunoglobulins (IgA, IgG, IgD, IgE, IgM) secreted by lymphocytic series cells, i. e. B-lymphocytes, plasmoblasts, and plasmocytes. The second group were the traits reflecting N-glycan levels in proteins (transferrin, haptoglobin, etc.) primarily secreted by hepatocytes, i. e. liver cells. The third group were the traits reflecting N-glycan levels in proteins secreted by both B-lymphocytes and their descendants and hepatocytes. The fourth group included the traits that were not classified. The classification was based on the data on glycoprotein presence published in (Clerc et al., 2016), where the authors evaluated the contribution of each N-glycoprotein into the N-glycome of human blood plasma

The loci and the associated traits in the network may be visually divided into two partially overlapping subnetworks. The first subnetwork is formed by loci containing ST3GAL4, B3GAT1, MGAT5, HNF1A, FUT3/FUT6, FUT8, and SLC9A9 genes. This subnetwork is associated with N- glycans linked to N-glycoproteins secreted into the blood stream by liver cells. Most of these traits reflect levels of trior tetraantennary N-glycans absent in immunoglobulins. This network includes the HNF1A locus encoding a hepatocyte transcription factor. The HNF1A locus, is associated with the same traits as the FUT3/FUT6 locus, which agrees with the role of HNF1A in fucosyltransferase expression regulation proved earlier (Lauc et al., 2010a). The results obtained make it possible to pose a hypothesis that this gene subnetwork regulates glycosylation processes in hepatocytes. The role of candidate genes from this network is to be tested in liver cells, e. g., hepatocytes, or cells close to them, e. g., HepG2 cell line.

The second subnetwork is formed by loci containing FUT8, FUT6/FUT3, SLC9A9, IKZF1, MGAT3, RUNX3, SMARCB1/DERL3/CHCHD10, B4GALT1, ST6GAL1, and IGH/TMEM121 genes. These loci are associated with N-glycans linked to immunoglobulins secreted into blood stream by lymphocytary series cells. In addition, it was shown in GWAS studies of N-glycan levels in IgG that these loci are associated with IgG N-glycosylation (Shen et al., 2017; Klarić et al., 2020). Since IgG is the most common glycoprotein in blood plasma, it can be hypothesized that candidate genes from this network regulate N-glycosylation processes in B-lymphocytes and their descendants. The role of candidate genes from this network should likely be tested in antibody-producing cells and cells close to them.

The role of transcription factor IKZF1 in FUT8 expression regulation in the lymphoid line GM12878 was proved in (Klarić et al., 2020). Furthermore, IKZF1 knockdown resulted in increased fucosylated protein level, which proves the role of transcription factor IKZF1 in protein fucosylation regulation as a result of in vitro experiment.

The GWAS approach was used to identify a total of 16 loci, and associations of 15 of them were confirmed in independent samples. An in silico study was performed for 15 confirmed loci, and 20 candidate genes were suggested. As a result of in vitro experiments, the role of transcription factor IKZF1 in protein fucosylation regulation and the role of HNF1A in fucosyltransferase expression regulation were proved. The role of transcription factor RUNX3 in N-glycosylation regulation was confirmed by targeted genome editing (using CRISPR-dCas9 system) in cell lines VPR-dCas9 and KRAB-dCas9 HEK-293F secreting IgG into the environment. Comparison of the IgG N-glycosylation profile with the non-modified control cell line showed that increased RUNX3 gene expression leads to a significant reduction of galactosylated structures with a further increase in agalactosylated structures (Mijakovac et al., 2022).

## Conclusion

The results of GWAS studies of N-glycan levels in blood plasma proteins confirm the understanding of N-glycosylation of human blood plasma proteins as a complex process controlled by genes involved in various biological pathways and expressed in various tissues. The candidate genes suggested as a result of a large-scale in silico investigation (Sharapov et al., 2019) of the confirmed loci make it possible to pose functional hypotheses on the mechanisms underlying the effect of the discovered loci on N-glycosylation of blood plasma proteins. These hypotheses will be of use in the planning of in vitro and in vivo molecular genetic studies of glycome and its role in pathogenesis of socially and economically important human diseases. The results of the performed in vitro experiments solidify the scientific credence of functional hypotheses with regard to candidate genes suggested using the GWAS approach.

There are several development trends for human population glycogenomics. Larger-scale GWAS studies into N-glycan levels using larger samples will be performed. New functional genomic data applicable to studying N-glycosylation processes will be available, which, combined with the GWAS results, will make it possible to identify more loci and potential N-glycosylation regulators. The application of the GWAS approach in glycosylation regulation studies is currently restricted to the analysis of the total N-glycome of blood plasma and N-glycome of IgG and transferrin. The development of N-glycome profiling technologies will expand the variety of proteins, the individual N-glycosylation profiles of which will be studied. On the other hand, highthroughput technologies for N-glycome profiling in other human tissues are likely to emerge. The advancements listed above will make it possible to better understand N-glycosylation regulation in human proteins and through that determine the role of glycosylation in pathogenesis of glycome-associated diseases and boost the development of new methods for prediction, prophylaxis, diagnostics and management of these diseases.

## Conflict of interest

The authors declare no conflict of interest.
